# A Low-Cost Breath Analyzer Module in Domiciliary Non-Invasive Mechanical Ventilation for Remote COPD Patient Monitoring †

**DOI:** 10.3390/s20030653

**Published:** 2020-01-24

**Authors:** Antonio Vincenzo Radogna, Pietro Aleardo Siciliano, Saverio Sabina, Eugenio Sabato, Simonetta Capone

**Affiliations:** 1 Institute for Microelectronics and Microsystems, National Research Council (CNR-IMM), Campus Ecotekne, Str. Prov. Lecce-Monteroni km 1.2, 73100 Lecce, Italy; pietro.siciliano@le.imm.cnr.it; 2 Institute of Clinical Physiology, National Research Council (CNR-IFC), Campus Ecotekne, Str. Prov. Lecce-Monteroni km 1.2, 73100 Lecce, Italy; sabina@ifc.cnr.it; 3 “A. Perrino” Hospital, Pulmonology Ward, 72100 Brindisi, Italy; sabatoeugenio@gmail.com; 4 Institute for Research on Population and Social Policies, (CNR-IRPPS), 72100 Brindisi, Italy

**Keywords:** COPD, exhaled breath, noninvasive ventilation, sensors, patient monitoring

## Abstract

Smart Breath Analyzers were developed as sensing terminals of a telemedicine architecture devoted to remote monitoring of patients suffering from Chronic Obstructive Pulmonary Disease (COPD) and home-assisted by non-invasive mechanical ventilation via respiratory face mask. The devices based on different sensors (CO_2_/O_2_ and Volatile Organic Compounds (VOCs), relative humidity and temperature (R.H. & T) sensors) monitor the breath air exhaled into the expiratory line of the bi-tube patient breathing circuit during a noninvasive ventilo-therapy session; the sensor raw signals are transmitted pseudonymized to National Health Service units by TCP/IP communication through a cloud remote platform. The work is a proof-of-concept of a sensors-based IoT system with the perspective to check continuously the effectiveness of therapy and/or any state of exacerbation of the disease requiring healthcare. Lab tests in controlled experimental conditions by a gas-mixing bench towards CO_2_/O_2_ concentrations and exhaled breath collected in a sampling bag were carried out to test the realized prototypes. The Smart Breath Analyzers were also tested in real conditions both on a healthy volunteer subject and a COPD suffering patient.

## 1. Introduction

Chronic respiratory failure is a serious pathological condition characterized by reduced efficiency of respiratory function; the lungs are not able to ensure adequate oxygenation of the arterial blood (hypoxemia) and/or to prevent CO_2_ retention (hypercapnia) [1]. The main condition that commonly leads to chronic respiratory failure is Chronic Obstructive Pulmonary Disease (COPD), an umbrella term used to describe progressive and not fully reversible lung disease involving emphysema (damage to the air sacs in the lungs), chronic bronchitis (long-term inflammation of the airways), and refractory (non-reversible) asthma [2]. The primary cause of COPD is cigarette smoking and/or exposure to tobacco smoke; other causes include air pollution, infectious diseases, and genetic conditions [3]. COPD represents an important public health challenge and it is a major cause of chronic morbidity and mortality throughout the world. Indeed, it is currently the 4th leading cause of death in the world but it is projected to be the 3rd leading cause of death by 2020 due to population ageing [4]. The impact of COPD on National Health Services (NHSs) in terms of cost and patient management is huge. The most critical issue is preventing the recurrence of acute exacerbations of COPD (ECOPDs), i.e., the episodes of worsening of symptoms, due to various factors, the most common being respiratory tract infections, which lead to hospitalization and increased mortality [5,6,7].

Noninvasive ventilation (NIV) refers to the integrated therapeutic treatment based on mechanical ventilators that assist (or substitute) breathing, using either pressure or volume control, with the help of a respiratory face mask; such ventilatory support improves pulmonary gas exchange and rest compromised respiratory muscles sufficiently to recover from the fatigued state [8,9]. This therapy has markedly increased over the past two decades in COPD treatment in replacement for invasive ventilation (endotracheal tube or tracheostomy tube), becoming a fundamental beneficial tool in patient management at home [10,11,12,13]. 

However, NIV administration in home setting gives rise to other problems related to domiciliary complex care of vulnerable patients under constant risk of sudden respiratory decompensation, as well as to health service management. Hypoxemia in COPD patients is due to ventilation/perfusion (V/Q) mismatch resulting from progressive airflow limitation and emphysematous destruction of the pulmonary capillary bed [14]; supplemental oxygen therapy is often used to manage hypoxemia but prolonged treatments are debated for the risks related to an inappropriate administration [15,16,17]. Patients with end-stage COPD frequently develop chronic hypercapnic respiratory failure associated with end-of-life; long-term NIV to treat chronic hypercapnic respiratory failure is still controversial in severe Chronic Obstructive Pulmonary Disease (COPD) patients [18]. 

The organization and cost of services for long-term domiciliary NIV assistance of patients are charged to NHSs; accurate individual titrations and frequent specialized technical support for adjusting ventilator setting and patient-ventilator asynchrony are required in order to optimize ventilation and minimize side effects. Patients and their families often feel alone in coping with daily ventilo-therapy without any medical monitoring. There is no integrated program of intervention on the territory aimed at ensuring proper patient assistance in the different COPD stages (mild, moderate, severe, end-stage) and supporting clinical decision-making in the successful management of domiciliary NIV [19].

Arterial-blood gas (ABG) test, measuring the amounts of arterial CO_2_ and O_2_, is the gold standard technique, but it is relatively invasive [20]. There is a growing interest in applying non-invasive technologies that could measure physiological parameters, as end-tidal CO_2_ (capnography) and blood oxygen saturation level SpO_2_ (oximetry) [21,22,23]. However, for domiciliary NIV, capnograph is an optional module of the mechanical ventilator, not included in the kit supplied to home assisted COPD patients. On the other side, most common oximeters do not allow SpO_2_ monitoring because they have no communication port or data storage and generally they are not provided to patients. There is, hence, the need to develop telemedicine systems for a remote monitoring of respiratory variables and patient surveillance under NIV [24,25,26,27]. The most diffused telehealth services are based on tele-assistance (teleconsultation, videoconference, etc.), but the most needed are those based on sensor technologies for monitoring the main physical variables of interest. Anyway, at present in both approaches home care programs controlled by telemonitoring are relatively rare [28,29,30,31,32]. The cost-benefit balance for telehealth technologies is debated; clinicians express negative generic advices and are generally skeptical about the effectiveness of such technologies, whereas patients were broadly in favor of devices that feel them supported in manage their disease [33]. A survey of the available technologies for the remote monitoring of Chronic Obstructive Pulmonary Disease (COPD) patients together with the future challenges are given by Tomasic et al. [34].

In this work we report the development of a low-cost breath analyzer implementable as external and independent module to almost any type of modern ventilator via compatible connections to expiratory line of bi-tube breathing circuit. We extended the description of the developed device presented at International Conference on IC Design & Technology held on 4th–6th June 2018 at Otranto, Italy (ICICDT 2018) [35]. The module allows continuous remote measurements of exhaled CO_2_, O_2_, and Volatile Organic Compounds (VOCs), as well as relative humidity and temperature (R.H. & T) during an NIV therapy session. A bundle of significant variables of exhaled breath are monitored. The module can be exploited to check continuously the status of hypoxemia and/or hypercapnia, avoiding invasive frequent blood draws needed for standard hemogasanalysis. As novelty, the information on the current health status, carried by exhaled VOCs, is traced through chemoresistive gas/VOC sensors.

The aim is to develop a system complementary to long-term domiciliary NIV for remote patient monitoring. The temporal tracings of all the sensor signals are sent to an ICT platform (OMNIACARE^TM^ eResults srl, Italy) [36] allocating a cloud storage. The clinicians can visualize the sensor tracks by connecting to the configured platform, use this auxiliary information to assess outpatient health condition, and be supported in making clinical decision for a successful management of domiciliary NIV.

## 2. Materials and Methods

### 2.1. Rationale

In the initial stage of the device design, the authors collaborated with specialist doctors; pulmonologists were asked to describe the main problems of home management of COPD patients and to express what were the not yet satisfied needs of technological innovation from a medical point of view. By these interactions and on the basis of physiological/clinical considerations, the main specifications of the device and the physical variables to be monitored, i.e., exhaled carbon dioxide/oxygen (CO_2_/O_2_) concentrations, relative humidity and temperature (R.H. & T), were defined.

**Figure 1:**
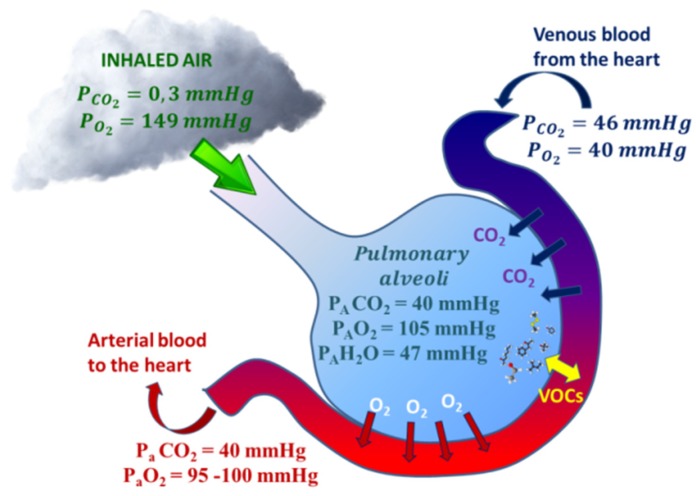
Respiratory gases (CO_2_ and O_2_) and Volatile Organic Compounds (VOCs) exchange within alveoli. CO_2_/O_2_ exchanges are approximated by the gas alveolar formula: P_A_O_2_ = (P_B_ − PH_2_O) × F_i_O_2_ − (P_A_CO_2_/R), where P_B_ is the barometric pressure, P_A_H_2_O is the water vapor pressure (usually 47 mmHg), F_i_O_2_ is the fractional concentration of inspired oxygen, and R is the respiratory quotient, dependent on metabolic activity and diet and is considered to be about 0.825). VOC exchanges depend on blood-air partition coefficients (λ_b:a)_.

Exhaled O_2_ and CO_2_ concentrations are correlated to alveolar gas exchanges and partial pressures of O_2_ and CO_2_ in the arterial blood (P_a_O_2_ and P_a_CO_2_). Alveolar equilibrium values (P_A_O_2_ and P_A_CO_2_) lie between those of the blood vessels and inhaled air and they can be measured by evaluating the values at the end-tidal of exhalation [37] (Figure 1). By monitoring end tidal O_2_ and CO_2_, it is thus possible to check continuously the status of hypoxemia and/or hypercapnia; the frequency of invasive hemogasanalysis could be reduced only to the necessary cases [38]. Exhaled O_2_ offer complementary information to blood oxygen saturation level (SpO_2_); SpO_2_ is expressed as a percentage of the maximum amount of oxygen that hemoglobin in the blood can carry and it is commonly measured by pulse oximetry. Unlike SpO_2_, exhaled O_2_ monitors the actual ventilation; exhaled O_2_ and CO_2_ are jointly good indicators of respiratory exchanges [21].

Whereas the respiratory cycle of CO_2_ and O_2_ gases is relatively straightforward and understood, the presence in exhaled breath of thousands of additional Volatile Organic Compounds (VOCs) is a powerful source of knowledge that reflects both environmental exposures (exogenous compounds) and relevant systemic and internal metabolic processes (endogenous compounds). Endogenous VOCs have been proposed to be useful as preclinical biomarkers of various undiagnosed diseases including lung cancer, breast cancer, and cardio-pulmonary disease [37,39,40]. Currently, there is a huge growing interest in Breathomics, the emerging metabolomics study of exhaled air that aims to find patterns of health-related VOCs. Breath analysis has not yet translated to clinical practice due to some critical issues not related to concept validity but to the need of more research to achieve fundamental goals as standardization of breath sampling protocols, validation by longitudinal multi-center clinical trials, understanding of the pathophysiologic origins of these VOCs by the biochemical pathways involved in disease development. A lot of efforts are devoted to get technological advances developing novel analytical and sensing platforms. Despite the difficulties in implementing breath-based diagnostics in daily clinical practice, scientific community believe that breath analysis will realize its long-held potential becoming a revolutionary tool in personalized medicine [40,41,42,43]. Breathomics studies applied to COPD showed distinct patterns of exhaled volatiles in COPD patients supporting the potential use of VOCs in discriminating COPD subjects and healthy controls as well as in identifying clinically relevant COPD subgroups [44,45,46]. Results published in literature demonstrated that Breathomics can be exploited in: (a) predicting inception of asthma or chronic obstructive pulmonary disease, (b) inflammatory phenotyping, (c) exacerbation prediction, and (d) treatment stratification [46,47,48,49].

Although the various forms of mass spectrometry, mainly gas chromatography-MS (GC-MS), proton transfer reaction-MS (PTR-MS), selected ion flow tube-MS (SIFT-MS) [50], provide information on exhaled substances with molecular identification and quantification, other more flexible, cheap and functional sensing platforms are electronic noses (e-noses). Such devices fingerprint the ensemble of exhaled VOCs (called human exhalome) in terms of gas/VOC sensors response patterns; their challenge is to become a screening non-invasive technique supporting gold mass spectrometric techniques [40,41,51,52,53,54]. 

Here the rationale is that e-noses could detect specific exhalome profiles associated with exacerbation states of COPD, that seem to be commonly caused by respiratory infections of viral or bacterial origin; the risk is higher in mechanical ventilated patients so that ventilator-associated pneumonia (VAP) is a known common phenomenon. Since many exhaled Volatile Organic Compounds (VOCs) may come from potentially pathogenic microorganisms metabolism [55,56,57,58], e-noses can be used for distinguishing between viral, bacterial, and non-infectious causes of exacerbations. Numerous literature reports on pilot studies based on e-noses analysis of exhaled breath to diagnose VAP [59,60,61,62,63,64]. This is important in order to reduce the over-prescription of antimicrobials for bacterial respiratory tract infection. 

Since breath is relatively warm and humid, R.H. & T are also important parameters to be measured in the exhaled air mainly as interference factors for infrared CO_2_ sensors and gas/VOC sensors based on semiconducting metal oxides (MOX). Recently, evaluation of the exhaled breath temperature (EBT) has been suggested as a new method to monitor pathological processes in asthma, chronic obstructive pulmonary disease, and other respiratory diseases [34,65,66]; novel respiratory rate monitor based on exhaled humidity has been also proposed [67].

### 2.2. Design Consideration

The Breath Analyzer Module was designed for a continuous breath-by-breath remote acquisition of signals from a set of sensors exposed to exhaled breath during long-term home-based NIV therapy [35]. The sensor toolbox was expanded including a small electronic nose (e-nose) based on 3-sensors array based on Metal OXide sensing materials (MOX) sensitive to exhaled Volatile Organic Compounds (VOCs) produced by ongoing internal biochemical processes of human metabolism. All the sensors (i.e., CO_2_, O_2_, R.H. & T, and MOX-based sensors) have been selected from the range of products available on the market based on sensing characteristics, technological quality, low power consumption, price considerations, and compatibility with medical applications.

**Figure 2:**
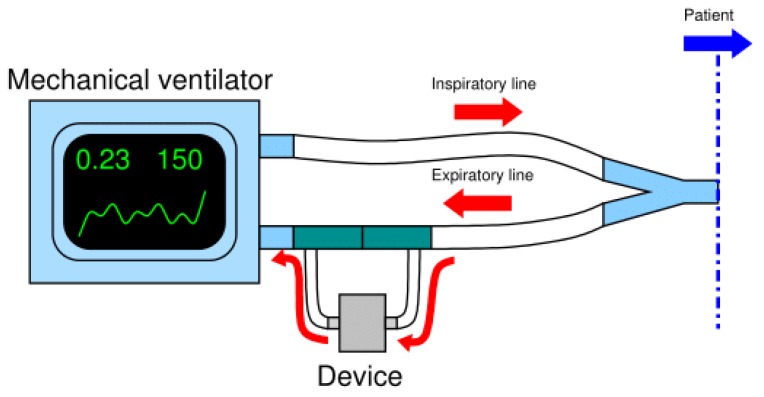
Scheme of the connection of the device to the expiratory line of the bi-tube breathing circuit.

The next challenge was to find the right setup of the system made by the mechanical ventilator and the breathing circuit in order to allow the connection of our monitoring device without causing any leakage alert in the ventilator, hence ensuring its normal operation in safety conditions for patient. The Breath Analyzer Module was connected sidestream to the expiratory line of a standard bi-tube patient breathing circuit upstream of the expiratory valve of the ventilator; a minor part of the exhaled air deviates from the mainstream into a gastight cell inside the device, then reentering in the mainstream. Such flow deviation configuration was implemented by two disposable tee-connectors, that are standard accessories of breathing circuits, making the device fully compatible with all mechanical ventilators set for bi-tube breathing circuit. Figure 2 schematizes the adopted connection of the breath analyzer module to the expiratory line of a bi-tube breathing circuit.

### 2.3. Hardware and Firmware Design

After a market inquiry, the following sensors accomplishing the designated tasks were selected:

(1) Infrared CO2 sensor: SprintIR™ by Gas Sensing Solutions Ltd., Cumbernauld, United Kingdom;
(2) Electrochemical O2 sensor: KE-25 by Figaro Engineering Inc., Osaka, Japan;
(3) Relative humidity and temperature: SHT75 by Sensirion AG, Staefa ZH, Switzerland;
(4) A dual (NOX-CO) sensor built with MOX technology: MiCS-4514 by SGX SensorTech, Corcelles-Cormondrèche, Switzerland;
(5) A VOC sensor built with MOX technology: AS-MLV-P2 by ams AG, Unterpremstaetten, Austria;

The choice of such commercial sensors was motivated by multiple considerations based on a compromise between sensing characteristics suitable for the specific medical application and mandatory requirements for a proof-of-concept of pre-commercial product (as miniaturization, low power consumption, and low cost). Table 1 summarizes the sensors main characteristic together with the main features addressing sensor selection for the Breath Analyzer Module.

**Figure 3:**
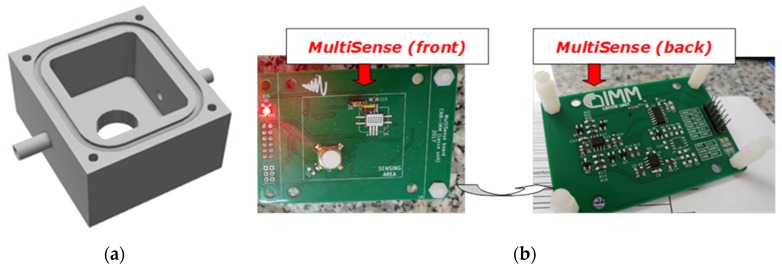
(**a**) 3D rendered model of the gas tight cell; (**b**) front and back views of *MultiSense* board.

The CO_2_ sensor was connected by its flow adapter cape to the deviating sidestream tube and to the inlet of a home-designed cell realized in Polyoxymethylene (POM); the O_2_ sensor was inserted through its threads into the screw hole on the cell top. Regarding to the other sensors, they are available only in Surface Mounting Devices (SMDs) and Pin Through Hole (PTH) packages, so that they required a custom designed electronic board, labelled *MultiSense*, that actuated also the necessary signal conditioning circuitry. The sensor cell was designed to be surface-mounted on the *MultiSense* Printed Circuit Board (PCB) and gas-tight by O-ring (Figure 3). 

**Figure 4:**
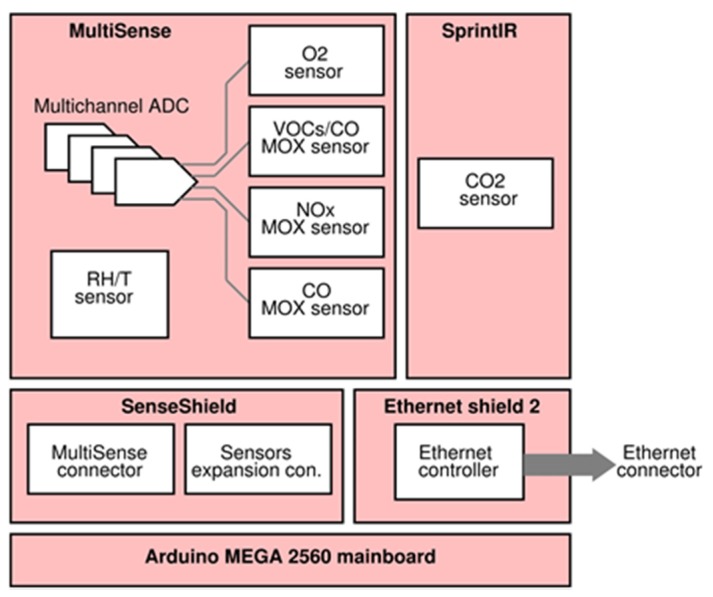
Device architecture.

**Figure 5:**
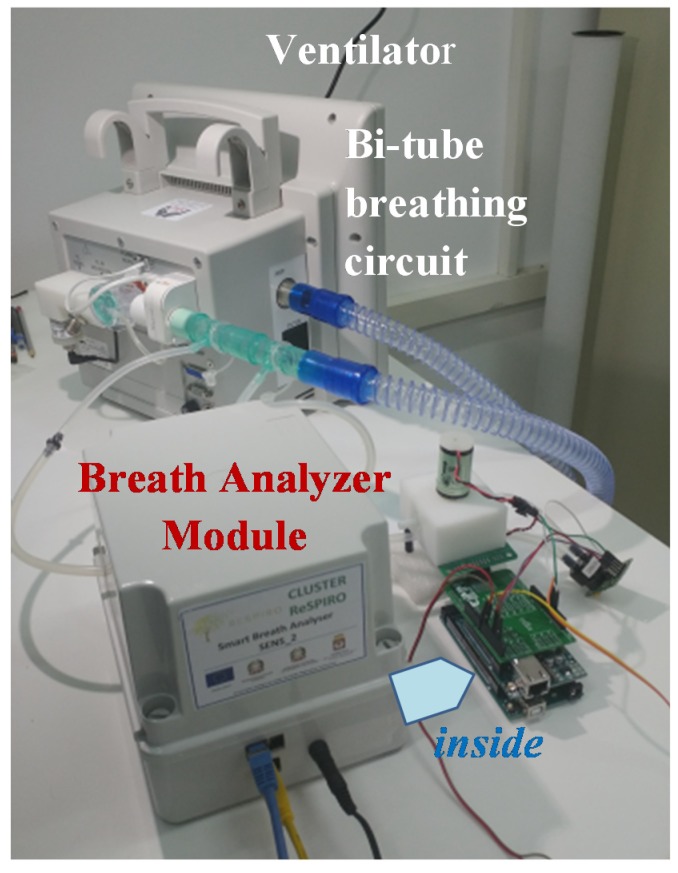
Realized unboxed prototype.

Since the target was to design a low-cost and easy to use tele-monitoring device, Arduino MEGA 2560 (Ivrea, Italy), equipped with Ethernet Shield v2 board for Arduino, was chosen as microcontroller due to its low price, low power consumption, and small size features; it also benefits of a simple and open source Integrated Development Environment (IDE) and a huge community support. In order to properly connect the *MultiSense* board with the Arduino platform, an additional electronic board, labeled *SenseShield*, was designed. Figure 4 and Figure 5 show, respectively, the device architecture and a picture of the realized prototype. 

**Figure 6:**
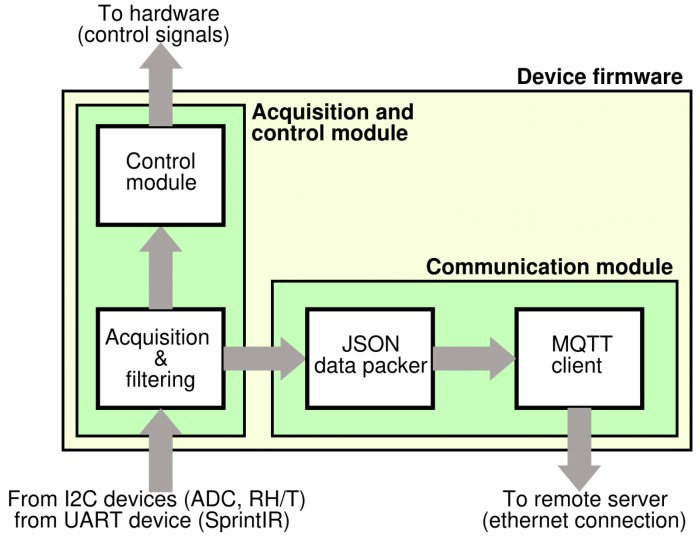
Device firmware overview.

**Figure 7:**
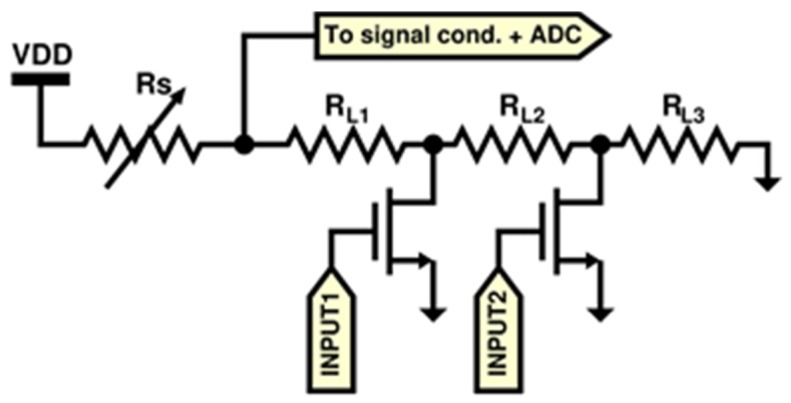
Auto-ranging circuit schematic.

Regarding the firmware, it was developed in two distinct modules: the first one implements sensor controls and signal acquisition, whereas the second one implements the communication and sending data to the ICT platform (Figure 6). The first firmware part is responsible for sensors setup, signal acquisition from digital sensors, analog-to-digital conversion and it also performs the sub-ranging algorithm for all of the MOX sensors to cover their large output resistance. A circuit schematic of the sub-ranging technique is depicted in Figure 7. Briefly, since the read-out circuit of the MOX sensor is essentially a voltage divider, the firmware selects, through a proper driving of the MOSFET switches, the load resistor closer to the sensor resistance thus determining a voltage ratio at the output of the voltage divider closer to VDD/2 (VDD system supply voltage). This is due to the fact that the closer they are two consecutive resistors of the voltage divider, the more sensitive the resistive reading is. 

The second firmware part implements the TCP/IP communication through Message Queue Telemetry Transport (MQTT), a lightweight communication protocol suitable for embedded and constrained systems. Since hardware resources are not capable of running secure standards like the X.509 certificates used in many secure protocols like TLS/SSL, data exchange from the device and the cloud server are not secured. However, this is not a remarkable problem because here MQTT messages do not contain sensitive data but only numerical variables without any reference to patient identity (pseudonymization of sensor raw signals).

**Figure 8:**
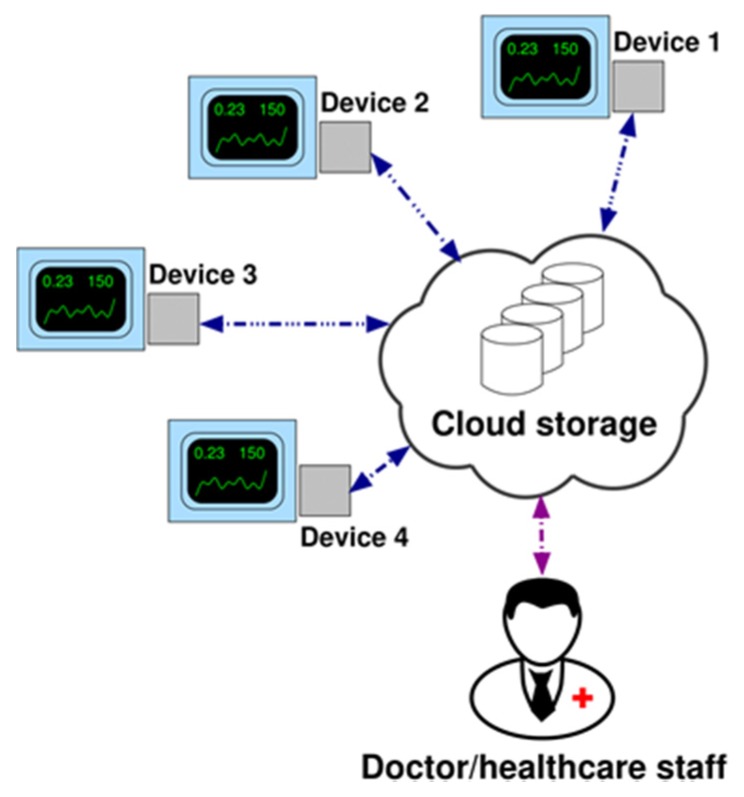
Network architecture (example with 4 devices).

Figure 8 illustrates the system network consisting of a certain number of devices, each of them connected to the ventilators of volunteer patients suffering from chronic respiratory failures and home-assisted by the health districts of Brindisi and Lecce in Italy. Thanks to this architecture and with the help of a control chart based server-side application, the doctor or the healthcare staff can control the monitored parameters to check the effectiveness of the therapy or any state of exacerbation of the disease. At present, 3 different prototypes have been produced in order to be used in the configured telemedicine system.

The ICT platform (OMNIACARE^TM^ eResults srl, Cesena, Italy) [36] that allocates the cloud storage integrates all the received aggregate data into a patient’s electronic medical record (EMR); in such EMRs other clinical parameters related to COPD patients were stored in digital format, allowing an integrated overview of the patient therapeutic path and its outcome. 

## 3. Results and Discussion

A preliminary characterization test of the Smart Breath Analyzer device was carried out in Gas Sensor Lab of CNR-IMM in Lecce and the aim was to verify the correct operation of the O_2_ and CO_2_ sensors, simulating real gas concentration ranges of end tidal values for exhaled O_2_ and CO_2_ corresponding to various levels (severe/moderate/mild) of hypoxemia and hypercapnia as well as basal conditions (normoxemia/normocapnia) [14,18] (Table 2). Exhaled air contains less oxygen and more carbon dioxide, it is also saturated with water vapor. Water vapor at body temperature exerts a partial pressure of 47 mmHg. Since the total pressure is always 760 mmHg and the relative percentages of the other gases do not vary, their partial pressure decreases. The O_2_/CO_2_ concentrations in % were hence converted considering the following formula:

(1) $$\;P_{gas}=\%gas\cdot \left(P_{atm}-P_{H_{2}O}\right)$$

where $P_{atm}=760\;\mathrm{mmHg}$ and $P_{H_{2}O}=47\;\mathrm{mmHg}$.

**Figure 9:**
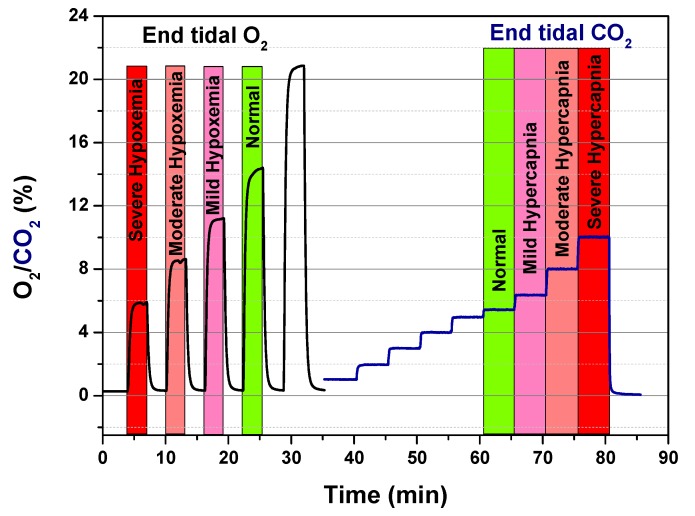
Calibration runs for O_2_ and CO_2_ sensor.

The tests were carried out with a gas-mixing station connected to different mass flow controllers (MFCs) and a MFCs multichannel unit to set the total gas flow and control the O_2_/CO_2_ concentrations in nitrogen through the dynamic dilution method. The total flow was set to 100 mL/min in dry condition. In Figure 9, the two sensor signals O_2_/CO_2_ (in %) vs. time during the related gas-sensing tests were reported as acquired by a developed device.

Further preliminary functional tests were carried out in laboratory by a healthy volunteer disconnected from the ventilator for safety reasons; without the use of facial respiratory mask the volunteer was asked to inhale through the nose and exhale through the mouth into the respiratory tube. Such tests (reported elsewhere [35]) proved the correct acquisition of all the sensor signals by the Breath Analyzer Module via PC serial port. 

**Figure 10:**
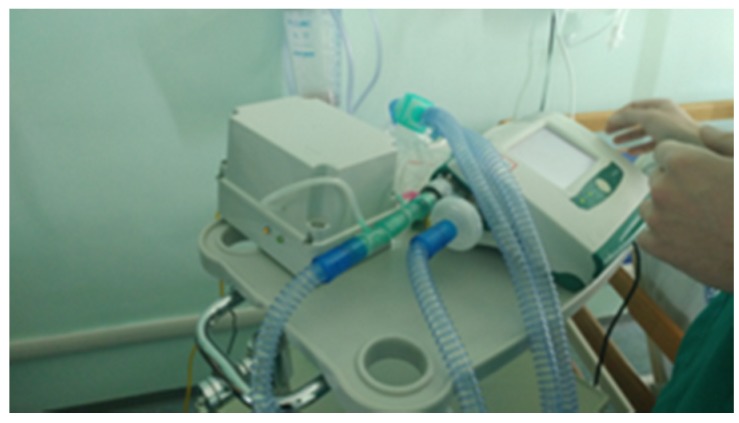
Breath Analyzer Module connected to bi-tube breathing circuit in real operating condition during clinical experimentation in hospital setting.

Next, after approval by the hospital Ethics Committee, experimentation on patients in a hospital setting was started. In particular, the device was tested in real conditions on a first volunteer patient during his NIV sessions with supplemental oxygen therapy at the pneumology unit of Brindisi hospital, Italy. Prior to participation in the study, the subject provided written, signed, informed consent. The Breath Analyzer Module was inserted, as configured in Figure 2, side-stream to the expiratory flow of a bi-tube breathing circuit connected to a mechanical ventilator (Figure 10); two water traps (not shown in photo) were also inserted in the breathing circuit with tube extensions thus increasing the length of a standard bi-tube configuration. 

The main aim of this first clinical experimentation was limited to verify the functioning of the implemented firmware for sensor acquisition in real operating condition during NIV. For this reason such initial tests were carried out in local without sending the data to developed remote server architecture, in order to differentiate the control of a correct data acquisition to the possible problems that may occur in data transmission; the data were transmitted to the serial port of a notebook computer via device USB port. 

**Figure 11:**
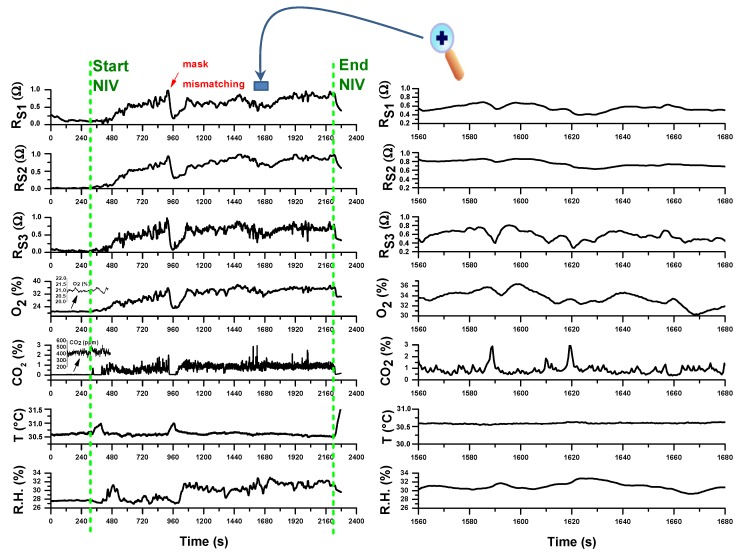
(**left**) Traces of all the sensor during a noninvasive ventilation (NIV) session with supplemental oxygen therapy; (**right**) magnification of a temporal segment during the trace recording. The vertical dotted green lines indicate the start and the end of the NIV session. The measurements refer to the first day of hospitalization of a Chronic Obstructive Pulmonary Disease (COPD) patient due to an exacerbation event.

Figure 11 reports the temporal registration of all the sensors tracings during a NIV session with supplemental oxygen therapy on a first volunteer COPD patient. The acquired raw signals in resistance of MOX-based sensors (R_S1_ from AS-MLV-P2 VOC sensor by AMS AG; R_S2_ and R_S3_ from dual NO_X_-CO sensor MiCS-4514 by SGX SensorTech) were normalized to [0,1] to compare the sensor responses:

(2) $$R^{\prime }=\frac{R-R_{min}}{R_{max}-R_{min}}$$

As it can be observed, the sampling protocol, according to a firmware version where all the sensors are sampled simultaneously in parallel with a sampling rate of 1 measurement at 500 ms, works correctly. All the gas sensors (CO_2_/O_2_ & VOCs sensors), each with its own response time, showed signal modulation enveloping one or more respiratory acts, but of course due to the device connection configuration not close to the respiratory face mask, it was not possible to deduce the end tidal part of an exhalation.

As expected, before NIV starting O_2_ and CO_2_ concentrations were at normal levels of inhaled air (20.89% for O_2_ and about 400 ppm for CO_2_); during NIV CO_2_ levels increases from inspiratory values to higher values of exhaled CO_2_. However, due to the position of the device at the end of the expiratory line of the breathing circuit, whose configuration can change according to specific therapeutic requirements, the CO_2_ end tidal values of the expired CO_2_ dilute not only due to physiologic dead volume but also to the dead volume of breathing circuit. Moreover, the expiratory reserve volume (ERV) is reduced in a COPD patient (typically about 500 mL), so that the measured CO_2_ is the result of more diluting exhalations in the overall dead volume. Although such limitations, the CO_2_ track is full of significant related-to-capnia physiologic information that can be easily extracted after a calibration with an additional standard capnograph sidestream connected to respiratory line (between the face mask and the Y-adapter of the bitube). This will be the next work activity.

In the particular case of this experimentation, after NIV start, the O_2_ concentration increases from inhaled O_2_ value to higher levels compatible with supplemental oxygen therapy. However, it can be observed according to respiration physiology that O_2_ decreases as CO_2_ increases in correspondence of exhalation. The O_2_ track is again full of significant related-to-hypoxemia physiologic information especially in NIV without supplemental oxygen therapy, just to support the eventual clinical decision for a supplemental O_2_ and its duration and administration modality. The correlation of exhaled O_2_ by the device with SpO_2_ values measured continuously by a pulse oximeter during NIV will be anyway a necessary step to validate the method. 

The signals from MOX-based sensors also vary during NIV accordingly both to O_2_ variation and exhaled VOCs content indicating a promising sensitivity to exhaled VOCs, especially for R_S3_ followed by R_S1_ and R_S2_ sensors. Of course a case-control study of COPD patients with and without pneumonia has to be planned and carried out in order to test if a statistical analysis of 3-sensors array data may be a tool to predict ventilator-associated pneumonia or an exacerbation condition. 

Relative humidity (R.H.) content in exhaled air is typically high (≈90%) and an increase of R.H. was indeed registered during the NIV session compared to the value before the start. However, due to the specific extended version of the used breathing circuit and to the use of water traps, the signal of the R.H. sensor did not saturate. Flow increases during NIV cause temperature decreases measured by T sensor. All the sensors registered an event of respiratory mask mismatching corresponding to patient’s state of agitation. 

**Figure 12:**
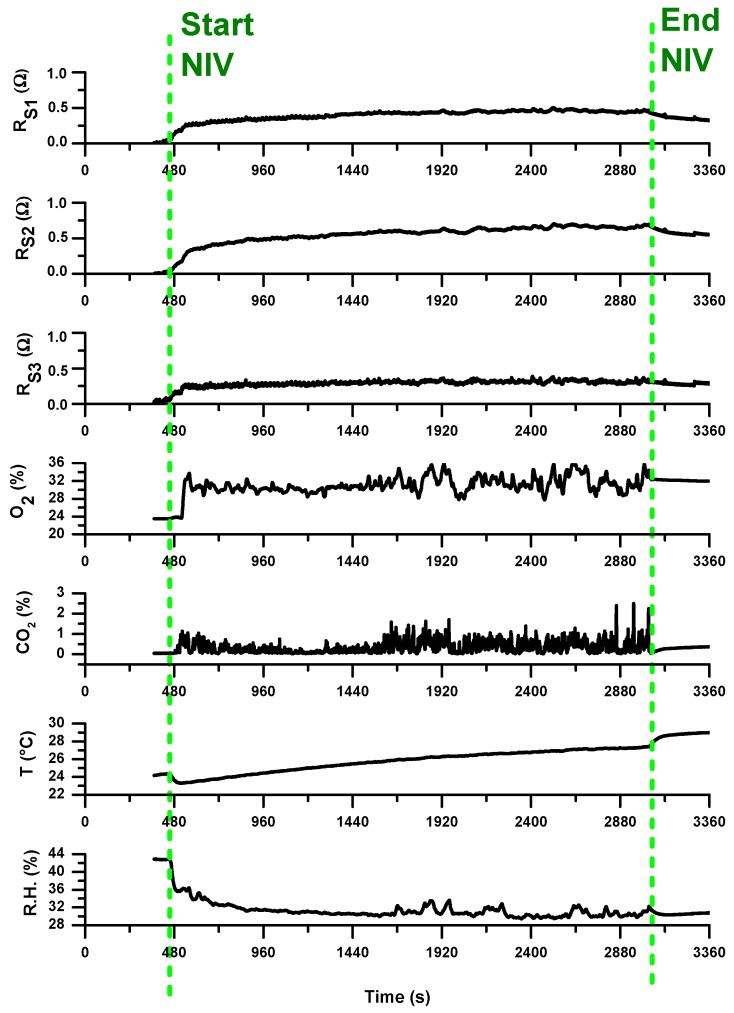
Traces of all the sensors during a NIV session with supplemental oxygen therapy; the vertical dotted green lines indicates the start and the end of the NIV session. The measurements refer to a hospitalization day following the first day of hospitalization (following that reported in Figure 11).

Analogous results were obtained monitoring over time the same patient during his period of hospitalization. Figure 12 reports the signals acquired from all the sensors during a ventilo-therapy session with supplemental oxygen corresponding to a hospitalization day following the first day of admission to hospital. 

As preliminary note, we can comment that all the 3-array MOX-based sensors showed a lower signal variation compared to the signals registered in first day. The characteristic VOC profile (breathprint) recorded during the initial phase of exacerbation at the time of hospitalization may be reduced in concentration or composition in the subsequent days of hospitalization. This may be a consequence of the pharmacological treatment to which the patient was subjected in the hospital to treat the exacerbation phase. The sensitivity of the sensors to such breathprint variations, probably linked to the presence of pathogens in the respiratory tract, is hence significant and promising for using e-nose in detecting the presence of bacterial infections in patients suspected of VAP. E-nose technology may become a rapid and non-invasive point-of-care tool for excluding the presence of pneumonia and, thus, withholding certain patients from receiving unnecessary antibiotic treatment.

**Figure 13:**
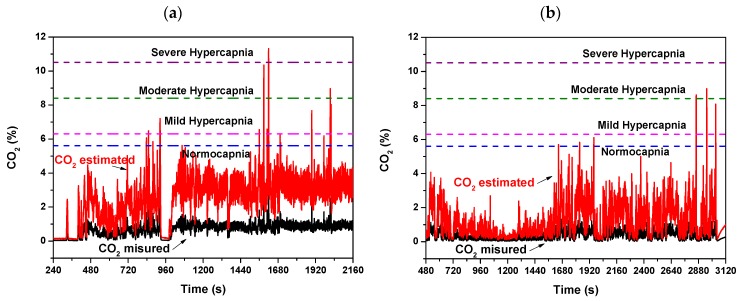

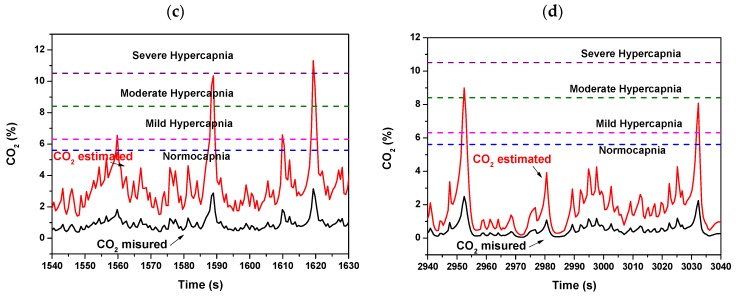
Exhaled measured and estimated CO_2_ concentration values monitored during a NIV session of the patient (**a**) in the first day of hospitalization and (**b**) in a hospitalization day following the first day. (**c**,**d**) are respectively magnified views of (**a**,**b**). The estimated CO_2_ values are calculated from the measured CO_2_ by coarse estimate by considering the volumetric dilution effect due to the dead space of the breathing circuit and of the water trap.

Moreover, the comparison between the measured exhaled CO_2_ temporal tracks during the two NIV sessions of the same patient, i.e., in the admission day at the hospital and in a next day, led us to make further considerations. By considering the volume of respiratory circuit for the peculiar configuration of used in the NIV sessions of this COPD patient under treatment, as well as the typical exhaled volume in a respiratory act, a multiplicative factor for the measured CO_2_ that corrects the volumetric dilution of the exhaled CO_2_ due to the dead space of the used breathing circuit plus that of the water trap was calculated in first approximation. The estimated exhaled CO_2_ tracks reveal that, during the NIV sessions, mild/moderate hypercapnia levels, up to severe hypercapnia level in the first hospitalization day (Figure 13a), have been reached. The condition improves over time; indeed, in the following hospitalization day, lower CO_2_ estimated values within normocapnia with only some spikes up to mild hypercapnia (Figure 13b) were registered.

Although the estimate of exhaled CO_2_ values is coarse, this suggests that maintaining the device sidestream configuration to the expiratory tube but shifting it towards the mouthpiece immediately after the Y-shaped connector of the bitube, the additional dead spaces due to the breathing circuit would be minimized and the tidal CO_2_ measured by the device more accurately. This was not possible because the tubes were not detachable from the Y connector in the embedded version of respiratory bitube system used in hospital. In such positioning the calibration with a capnography, as discussed above, will complete the validation of the device as regards CO_2_. 

Analogous considerations could be done for O_2_; a positioning of the developed Breath Analyzer Module closer to the mouthpiece would allow also a more accurate measure of the exhaled O_2_. The measured exhaled O_2_ could be more easily related to normoxemia or hypoxemia status. The relevance in monitoring exhaled oxygen in clinical practice as decisional support to pre-oxygenation treatment is known, since SpO_2_ alone is not adequate for monitoring alveolar ventilation and hemogasanalysis is invasive [21,68,69,70]. A low-cost portable exhaled O_2_/CO_2_ analyzer is hence a valid candidate to satisfy such monitoring at patient bedside. 

## 4. Conclusions

A novel low-cost Breath Analyzer Module device for remote COPD patient monitoring during domiciliary non-invasive mechanical ventilation, was designed, realized, and tested. At present, 3 different Breath Analyzer Modules have been produced ready to be used as terminals of the configured telemedicine system. The device is multi-sensors based. It enhances the monitoring features of basic mechanical ventilators used in home NIV, providing sensors for O_2_/CO_2_ concentration, temperature and relative humidity in the exhaled air of COPD patients. 

The developed Breath Analyzer Module is also:

- Universal: it can be used as external module for any ventilator with bi-tube breathing circuit;
- Plug & Play: it requires only basic connections without configuration;
- Low-cost and highly customizable: it is based on low-cost hardware (Arduino);
- IoT-oriented: the device can communicate data over TCP/IP communication (wired).
- Flexible to further implementations: the system may be configured for advanced data processing in OMNIACARE hardware/software platform to support local healthcare staff to check the effectiveness of therapy.

Furthermore, the device including a small array of three chemoresistive sensors (electronic nose concept) based on MOX technology provides a fingerprint of the Volatile Organic Compounds (VOCs) pattern in the exhaled air. The integration of electronic nose (e-nose) technology in non-invasive ventilo-therapy and/or routine spirometry, is a strategic plan to bring this technology to ‘point-of-care’ for respiratory diseases, enabling the detection of ventilator-associated pneumonia. Breath analysis offers indeed a unique opportunity to retrieve relevant information on ongoing internal biochemical processes noninvasively, since a lot of volatile components are exchanged at cells/blood and blood/alveoli interface from alveoli are subsequently exhaled. Moreover, by considering that the results from the e-nose integrated in our Breath Analyzer Module could be also combined with those from consolidated analytical methods (as GC/MS), we believe that the realized point-of-care devices could be a useful tool providing new opportunities for monitoring the patient actual health status and discovering breath VOC markers characteristic of pulmonary and systemic conditions.

From first evidence of initial clinical experiments, all the acquired sensor traces are valid for a next data analysis and correlation with routine clinical examinations and clinical data. In particular, in relation to the 3-array chemoresistive sensors, suitable signals processing and statistical pattern recognition techniques have to be applied. Of course, an appropriate suitable interpretation of all the monitored parameters by a physiological point of view is required. At this aim a device positioning a closer to the respirator mask can certainly help in providing sensor profile outputs more similar to those of standard sidestream configuration. Further efforts will be devoted to check the reliability and robustness of the telemedicine system in relation to the communication protocol and data transmission. An extensive clinical experimentation is also necessary to validate the Breath Analyzer Module as well as train it on a sample population of patients in different COPD conditions. This stage is fundamental to build an intelligent software that, based on data analysis algorithms, provides indication about restoration of a correct respiratory function during NIV therapy. 

The developed system looks forward to an advanced evolution in which the Breath Analyzer Module is integrated into the ventilator or constitutes an optional additional external component; in this new scenario for patient monitoring system, it is the ventilator itself which becomes a terminal of the telemedicine system and transmits all the parameters recorded and displayed during home ventilation to an IoT platform. Manufacturers of mechanical fans may be interested in developing a ventilator model designed for inclusion into a remote monitoring system. 

## Figures and Tables

**Table 1:** Sensors characteristics

Sensor	Technology	Accuracy	Sensing Range	Power Consumption	Comm.	Sensor Main Features Addressing Sensor Selection for the Breath Analyser Module
SprintIR	NDIR with flow through adapter	±70 ppm +/− 5% of reading	0–20%	35 mW	UART	• Sensing range compatible with hypercapnia levels • Flow adapter cape with 1 inlet and 1 outlet compatible with sidestream connection to system • Application Note AN-128 for operating with Arduino
KE-25	Galvanic cell	±1% full scale	0–100%	N/A	Analog (voltage)	• Suitable for medical applications • Linear output voltage signal relative to percent oxygen • No external power supply required for sensor operation • Virtually no influence from CO_2_ • Threaded top suitable for connection to sensor chamber • Low cost
SHT75 (T)	Proprietary CMOSens^®^	±0.3 °C	−40 °C–123.8 °C	90 µW (average)	I2C	• High accuracy • Attractive price-performance ratio • Easy replaceability (pin-type version) • Fully calibrated digital output • Low power consumption • High-end version
SHT75 (RH)	Proprietary CMOSens^®^	±3.0%	0–100%			
AS-MLV-P2	Metal-Oxide	N/A	30 ppm–500 ppm (taken from CO sensitivity curve, T and RH not mentioned)	34 mW (heating element at 320 °C)	Analog (Resistance)	• Miniaturized MEMS (micro electromechanical system) devices • High sensitivity to VOCs (AS-MLV-P2 and MICS-4514 (RED)) • High sensitivity to NO_2_ (MICS-4514 (OX) to catch those exhaled NO molecules, known inflammatory marker, converted in NO_2_ • Very low power consumption • Surface Mounting Device (SMD) package compatible with Printed Circuit Board Assembly (PCBA) • Compact and simple front-end (conditioning circuit based on buffered voltage divider) • Low cost (10–20 €) • Scarce selectivity compensated by sensor array with cross-sensitivities
MiCS-4514 (RED)	Metal-Oxide	N/A	1 ppm to 1000 ppm (taken from CO sensitivity curve, 25 °C, 50% RH)	88 mW (heating element)	Analog (Resistance)	
MiCS-4514 (OX)	Metal-Oxide	N/A	0.05 ppm to 10 ppm (taken from NO_2_ sensitivity curve, 25 °C, 50% RH)	50 mW (heating element)	Analog (Resistance)	

**Table 2:** Alveolar partial pressure levels ($P_{O_{2}}$ and $P_{{\mathrm{CO}}_{2}}\;$ in mmHg) and alveolar concentrations (O_2_% and CO_2_% in percentage by volume) of oxygen and carbon dioxide in basal normoxemia/normocapnia and severe/moderate/mild conditions of hypoxemia/hypercapnia.

	$P_{O_{2}}$ **(mmHg)**	**O_2_ (%)**
Normoxemia	100	14.0
Mild Hypoxemia	60–80	8.41–11.22
Moderate Hypoxemia	40–60	5.61–8.41
Severe Hypoxemia	<40	<5.61
	$P_{CO_{2}}$ **(mmHg)**	**CO_2_ (%)**
Normocapnia	40	5.61
Mild Hypercapnia	45–60	6.31–8.41
Moderate HyperCapnia	60–75	8.41–10.51
Severe HyperCapnia	>75	>10.51

## Abbreviations

The following abbreviations are used in this manuscript:

VOC	Volatile Organic Compound
COPD	Chronic Obstructive Pulmonary Disease
ECOPD	exacerbation of COPD
NIV	Noninvasive ventilation
NHS	National Health Service
TCP/IP	Transmission Control Protocol/Internet Protocol
R.H. & T	Relative Humidity & Temperature
MFC	Mass Flow Controller
GC/MS	Gas Chromatography/Mass Spectroscopy
IoT	Internet of Things
MQTT	Message Queue Telemetry Transport
TLS/SSL	Transport Layer Security/Secure Sockets Layer
PCB	Printed Circuit Board
SMD	Surface Mounting Devices
PTH	Pin Through Hole
IDE	Integrated Development Environment
PC	Personal Computer
ICT	Information and Communication Technology
MOSFET	Metal Oxide Semiconductor Field Effect Transistor
